# Current Biochemical Applications and Future Prospects of Chlorotoxin in Cancer Diagnostics and Therapeutics

**DOI:** 10.15171/apb.2019.061

**Published:** 2019-10-24

**Authors:** Sbonelo Khanyile, Priscilla Masamba, Babatunji Emmanuel Oyinloye, Londiwe Simphiwe Mbatha, Abidemi Paul Kappo

**Affiliations:** ^1^Biotechnology and Structural Biology (BSB) Group, Department of Biochemistry and Microbiology, Faculty of Science and Agriculture, University of Zululand, KwaDlangezwa 3886, South Africa.; ^2^Department of Biochemistry, College of Sciences, Afe Babalola University, PMB 5454, Ado-Ekiti 360001, Nigeria.

**Keywords:** Cancer, Chlorotoxin, Diagnostics, Matrix metalloproteinase-2, Therapeutics, Tumor

## Abstract

Chlorotoxin (CTX) is a minute 4 kDa protein made up of 36 amino acid residues, commonly known for its binding affinity to chloride channels and matrix metalloproteinase-2 (MMP-2) of glioma tumors of the spine and brain. This property and the possibility of conjugating this peptide to nanoparticles have enabled its diverse use in various biotechnological and biomedical applications for cancer treatment, such as in tumor imaging and radiotherapy. Because of the fascinating biological properties CTX possesses, elucidating its mechanism of action may hold promise for the development of new and effective therapeutic drugs, as well as more sensitive and highly specific cancer-screening kits. This article therefore reviews the currently known applications of CTX and suggests diverse ways in which it can be applied for the design of improved drugs and diagnostic tools for cancer.

## Introduction

Worldwide and since ancient times, traditional medicine has been used in poor and rich communities alike for the prevention and treatment of numerous diseases, including cancer. Several cultures still remain dependent on both plants and animals for treating medical ailments owing to the natural bioactive compounds these possess and over the years, a substantial amount of bioactive compounds have successfully been isolated from plants.^1^ Several of these compounds have been made available to the public and more than 60% of them are currently in use as anti-cancer agents.^2^ Current research has, however, diverged slightly into exploring natural compounds from animals, such as peptides from venoms, which have been revealed as potent and promising candidates in drug discovery ventures.^3^ Alpha-bungarotoxin is among one of the very first venom peptides to have been discovered in the early 1960s from the elapid Taiwanese branded krait snake and is best known for its competitive binding affinity to certain subtypes of the nicotinic acetycholine receptors found in the brain; thus it is involved in countless applications in neuroscience.^4^ Its discovery has since then paved the way for several studies to take a closer look at snake venoms, with specific emphasis on their many contributions in the medical field, evident from the almost 40 000 articles published in the field yearly.^5,6^

Different studies have shown that venoms are non-toxic and therapeutic when administered at very low doses; concern is only raised once certain concentrations occur in excess.^7^ In the past, venoms were primarily used for hunting or as weapons of war and later for various medical conditions. In countries such as China, venom from toad skin was traditionally used to treat illnesses such as leukemia, while in other countries, such as India, snake venoms were used to extend life expectancy.^8,9^ These and other reasons, therefore, give credence to the saying; “One man’s poison is another man’s medication”.^10,11^ Although their presence has been acknowledged for years, little or no research has been conducted at the molecular level to understand the biochemical functions of these substances or the interactions that may be involved regarding their activity, toxicity or therapeutic effects.^12,13^ Lately, however, these peptides have been recognized as curious yet important sources of ‘lead’ structural templates for the design of new drugs.^14,15^

Venoms are now considered “gold mines” for insecticides, therapeutic agents and pharmacological substances owing to their different properties.^13,16^ Neurotoxic peptides are venoms that contain mucopolysaccharides, hyaluronidase, phospholipase, serotonin, histamine, enzyme inhibitors and proteins. These venomous peptides possess the ability to initiate or cause great damage to the nervous systems of both vertebrates and invertebrates by interacting with ion channels such as sodium, potassium, and chloride channels, which assist in regulating various ions required for normal cell functioning and other pathophysiological processes.^10,11^ Biomedically, venoms are now considered important because of the specific binding affinity they show with certain ion channels. Although their main function is to disrupt normal physiological processes, they have also been proven to be useful components in the development of drugs, explosive detectors and anti-venoms. In addition, their extreme and highly specific properties act as an advantage when they are used as biomedical drugs to target specifically vital organs and pathological pathways.^17,18^ Hence, this review will focus on the currently available biomedical applications of a venomous peptide called chlorotoxin (CTX) in the treatment of cancer with the use of imaging, radiotherapy, and nanotechnology-based applications. Furthermore, future use of CTX in the formulation and improvement of cancer drugs and diagnostic tools for early cancer screening is reviewed.^19-21^

## Chlorotoxin: A biological peptide from Leiurus *quinquestriatus*

CTX is a small venomous peptide that was first isolated in 1993 from *Leiurus quinquestriatus,* an Israeli scorpion. It forms part of a venomous family of insectotoxins, which contain different substances such as enzymes, toxins, non-protein inclusions and peptides.^22-24^ Studies have concentrated on one of its unique features, its size, which is small enough to diffuse or pass through membranes and penetrate deep internal tissues or firm tumors where other molecules such as antibodies cannot reach because of their naturally big size.^3^ CTX biologically binds to chloride channels on the surface of glioma cells and functions by blocking the influx and outflux of chloride ions, which are required for normal cell functioning. It has been postulated that its activity may lead to processes such as paralysis, a phenomenon the scorpion primarily uses for its survival by paralysing its prey for food, as well as protecting itself from predators.^22,25^ Since the protein is a member of the insectotoxin family, its insecticidal properties have been tested on different insects and organisms, such as the American cockroach and crayfish. Studies have shown that these toxins exert their biological effect by instigating motor control failure, which may then progress and eventually lead to paralysis.^25^ However, this occurrence has not been reported in mammals.^26^

CTX is made up of 36 amino acids with residues 1-9 and 30-35 identified as the most conserved in the sequence. Eight cysteines are present that make up four disulphide bonds, which stabilise the protein structure by reducing their conformational flexibility.^22,27^ Characterization of the protein secondary structure by nuclear magnetic resonance (NMR) spectroscopy has shown that amino acids 1-4, 26-29 and 32-36 form three β-sheets respectively, while the presence of a single and the only tyrosine residue in the sequence at the 29^th^ position is useful in enabling iodination.^24,27^ The 3D structure of CTX agrees with other short toxins such as charybdotoxin. Overlay of the two structures showed that both possess three disulphide bridges, while the fourth, made of Cys2-Cys19 in CTX, is replaced by two valines in charybdotoxin. CTX-like peptides such as AaCTX from *Androctonus australis,* BmKCTa from the *Buthus martenzii* scorpion and GaTx1 and GaTx2, which are both from the venom of the *Leiurus quinquestriatus* scorpion, have been isolated and have demonstrated similarity in their structures and functions.^24,28^ Sequence alignment of CTX and these CTX-like peptides showed that BmKCTa and GaTx1 have 67% and 64% similarity respectively. Interestingly enough, BmKCTa and GaTx1, as well as CTX, have been tested on ion channels other than chloride and have been shown to possess no functional activity on these ion channels except on chloride.^29^ This therefore leads to the conclusion that CTX and CTX-like peptides bind with high specificity only to MMP-2 of human astrocytoma cells, including STTG1 cells under non-physiological conditions, thus making them highly specific targets of MMP-2 during non-physiological states and good candidates for diagnostic tools.

For peptides to be stable, compact and considered useful in therapeutics, they should posses the following characteristics: (i) small molecular size; (ii) clear activity on ion channels; and (iii) containing at least three disulfide bonds.^25^ From all indications, CTX meets all the vital criteria of being a therapeutic peptide and is hence a good candidate in medical research owing to its bioavailability and ability to reduce target selectivity, which in turn reduces drug side effects such as drug resistance due to lack of specificity. Moreover, its membrane permeability and metabolic stability will account for lower cost of drugs.

During its discovery, as was observed in human glioblastoma multiforme (GBM) by Cheng and colleagues,^30^ it was shown that CTX inhibits the flow of chloride ions, actively binding to and blocking chloride ion channel-3 (CLC-3), whose activity is associated with the invasiveness of gliomas by allowing the flow of ions inside the cell for normal functioning.^30^ Recent studies have pointed out Annexin A2, a member of the Annexin family, a calcium-reliant phospholipid-binding protein family, whose primary function is repair of the plasma membrane as a molecular target for CTX. Annexin proteins also facilitate other processes such as the binding of anionic phospholipids and the regulation of different biological processes at the cellular level such as membrane segregation, which requires calcium ions (Ca^2+^),^31^ studies have, however, shown that developing effective therapies by blocking CLC-3 alone is not enough to cure GBM cancer. This has therefore prompted researchers to identify other molecular targets for CTX and hence, current functional studies have suggested matrix metalloproteinase-2 (MMP-2) as an interacting partner.^32^

MMP-2 is a protein that is upregulated in all tumor cells and possesses type IV collagenolytic activity.^32,33^ This protein is expressed as a 72 kDa zymogen that is activated by the removal of the pre- and pro-domain to an active 64 kDa mature protease.^34^ This metalloenzyme requires a zinc ion in its active site for catalytic activity.^35^ Under normal physiological conditions, it maintains tissue allostasis and degrades extracellular components that include basement membrane collagen and extracellular matrix (ECM) macromolecules such as fibromodulin, decorin, biglycan components and additional constituents such as fibronectin. The activation of MMP-2 is a vital process required by GBM for the ECM to degrade, resulting in the relocation of the cancer to distal parts of the body in a process called metastasis. Although a few researchers have suggested that MMP-1 plays a more important role than MMP-2 in the migration, remodelling, and invasiveness of GBM, it has been shown that MMP-2 plays a vital role in the virulent progression of cancer by contributing to three vital processes: angiogenesis, metastasis, and invasion.^36-39^ Angiogenesis is the normal developmental process of new blood vessels from already existing ones, which serves as an imperative factor in the wound-healing process and in the female reproductive cycle. In certain pathological states, this process initiates or promotes tumor formation in diseases such as cancer, diabetic retinopathy, hemangiomas and rheumatoid arthritis.^40^ Although the main role of MMP-2 seems to be the simple degradation of the ECM, recent studies have indicated that it is in fact a difficult process to understand.^41^ Cancer invasion is a process driven by cells or tissues, where they undergo a series of physical, cellular and molecular processes to aid cancer progression and in turn make themselves harmful to nearby cells.^42^ A series of mechanisms occur from within the tumor cell before it migrates to its surroundings to invade nearby cells. The proteolytic activity of MMP breaks down the physical barrier that inhibits cancerous cells from invading healthy or normal cells.^43^ During the pathological state, MMP-2 disrupts the cell membranes of normal cells during cleavage of the ECM. According to Eccles and Welch,^44^ a continuous supply of oxygen and blood is needed by newly-formed tumor cells and angiogenesis comes into play by promoting tumor growth. It is generally known that cancerous cells target and invade normal healthy cells and studies have confirmed that MMP-2 enables this process by playing a pivotal role in the progression of cancer to distal parts of the body from its point of origin through metastasis.^44^

## Cancer and its development

Cancer has become one of the most problematic diseases worldwide. Statistics show that tuberculosis, HIV/AIDS and malaria combined kill fewer people than cancer.^45^ GLOBACAN statistics have stated that in the year 2012, approximately 14 million new cancer cases were reported and this number was expected to increase to 24 million (including deaths) by the year 2030.^46-48^ Cancer development and its progression is a highly documented process that has been shown, in basic terms, to involve the irreversible genetic change or mutation of normal healthy cells to modified malignant cells.^49^ Three main genes play an important role in the initiation process of cancer: tumor suppressor genes, proto-oncogenes and the genes that are involved in DNA repair processes.^50,51^ Any mutation or deletion of the above-mentioned genes, as well as the over-expression of proteins such as epidermal growth factor receptor, leads to the disturbance of normal regulated cell growth and differentiation. Therefore, inhibiting proteins or enzymes that promote cancer stimulate strategies for designing more effective drugs to combat the disease.^52^ Thus, the use of small molecule inhibitors to treat diseases is now at its peak in biomedical research, mostly through the use of bioinformatics as a quick technique that is not limited to any type of technology or information that is mostly obtained even before molecular wet laboratory experiments are embarked upon.^53^ Ever since the introduction of bioinformatics, the development of inhibitors to treat diseases has improved over the years and this is considered one of the “success ventures” of the 21st century, as it has propelled scientific interest in the fight against cancer.^54-56^

Although researchers have unanimously agreed that cancer is initiated by multiple mutations, they have failed to address the main cause of this occurrence and the chromosomal abnormalities that are involved in cancer initiation processes. This usually occurs in unstable genome systems, resulting in the body becoming extremely sensitive to very minor changes such as chromosomal alterations within the cell.^57^ Unlike individuals with highly active immune systems with the ability to ward off cancer through cancer immune-editing processes, those highly susceptible to the disease, in most cases, have weak immune systems that fail to defend their bodies against cancer.^47,58,59^ The immune-editing process is sub-divided into three-course action: elimination, equilibrium, and escape. The first step (elimination) represents the process where the immune system detects and destroys viral and neoplastic-transformed cells in the body in a bid to prevent growth to the next phase of development, but this requires significant activity of both innate and adaptive immunity.^60^ The innate immune system is the first responder after receiving an alert of a growing tumor.^61,62^ Various components and elements that are involved in such processes have been identified; however, exact details pertaining to their functions still need further clarity.

In the second step, interferons are released at the tumor site, encouraging the production of chemokines or signalling proteins, thus calling the attention of the innate immune system to the tumor site.^63^ This leads to interleukin activation and the production of natural killer (NK) cells that set in motion a cascade of different interferon-dependent reactive processes, such as angiostatic, anti-proliferative and pro-apoptotic processes, as well as the activation of macrophages, which produce reactive oxygen and nitrogen intermediate products at a fast rate. These processes become harmful to tumors and ultimately destroy them. The tumors’ specific immunity enables the host immune system to recognise and completely eliminate tumors at a very early stage. However, it is possible that tumors are able to evade and survive this step. Hence, any variant that has escaped the elimination phase is destroyed in the equilibrium phase, which is the longest process in cancer immune editing. Cell variants may arise and exhibit different mutations, thereby causing the cancerous cell to escape the immune response. The equilibrium phase is therefore responsible for reducing the immunogenicity of such mutant cells. Tumors may, however, escape this phase again owing to growth in an immunologically unharmed environment, thus developing different approaches to escape both innate and adaptive responses. Such a “cancer escape” may be influenced by a number of different factors, including loss of tumor recognition by the immune system due to alteration or loss in anti-apoptotic and/or NK cell signals. This phase, nevertheless, requires additional research to elucidate how tumor cell immunogenicities are edited by the immune system.^64,65^

Once escape has taken place, tumorigenicity then follows, where tumors invade nearby cells until the cancer metastasizes to distal and vital organs of the body such as the lungs, liver, stomach and kidneys. This represents the most dangerous stage of cancer and is often referred to as the “end stage” because of the high amount of damage it causes to the organs of the body. This phase may be avoided but unfortunately, most cancers are only diagnosed during this stage, resulting in increased chances of mortality, mainly because neoplastic progression (abnormal growth) causes death.^44^ Nevertheless, not all tumor cells metastasize to distal parts of the body; some remain locally invasive.^44^ The early detection of both malignant and benign tumors through novel technologies as well as cancer treatment that specifically targets metastasis is therefore needed.^44^ CTX has shown promise in addressing this challenge in three ways: imaging, nanotechnology and radiology.

## Chlorotoxin in imaging cancerous cells

Tools for the molecular imaging of live cells and whole tissues are of vital importance when it comes to studying cancer, as well as formulating efficient and effective therapies for the disease. The presence of lymph nodes in cancer has now become a prime target in the study of cancer and possible therapeutics.^66^ The first node in the lymphatic basin, called the sentinel lymph (the tumor of origin), is usually targeted by oncologists at the first attempt in cancer treatment. However, visualising this node is problematic for most oncologists, since cancerous cells need to be differentiated from normal or healthy cells.^67^ This is quite important, as most current cancer drugs unfortunately lack specificity when administered and consequently affect conventional cells. An experiment designed by Stroud and colleagues saw CTX conjugated to Cy5.5 dye (CTX:Cy5.5) and administered to rats inflicted with tumors.^68^ Positive results from NMR experiments showed the presence of CTX in the Cy5.5 dye, owing to the affinity of CTX in binding to extracellular membranes of tumors, aiding the dye to reach tumor sites.^69^ Because of this, surgeons now have the ability to remove cancerous tumors without affecting normal cells. Pharmacological properties have also been improved by producing mono-labelled and cyclic peptides through bioengineering. This is achieved by removing Lys15 and Lys23 from natural CTX and substituting these two amino acids with either an alanine or arginine; the only lysine on position 27 is then left to produce the mono-labeled molecule.^24^

## Use of chlorotoxin with nanoparticles in cancer

Nanobiotechnology is a 21st century technology and scientific field under rapid development that involves two interdisciplinary aspects (nanoscience and biotechnology) that bring together the fields of bioengineering and advanced science, using nanostructured materials, especially nanoparticles (NPs), in applied medicine.^70,71^ Improvements in this field have drawn the attention of scientists to identify domains that can be hypothetically conjugated to NPs with the aim of developing low-cost effective drugs. NPs have rare properties that can be used positively in improving pharmacological and therapeutic agents when conjugated with drugs, instigating a faster and more precise delivery procedure.^72^ NPs are chemically synthesized materials having a length of less than 100 nm that have been used as drug delivery systems because of their large surface area, ultra-small size, high reactivity and stability, effective interaction with cells and catalytic power, as well as their solubility.^12,73^ Their surface area allows them to carry therapeutic drugs, while their small size gives them admission through very small membrane passage-ways and consequently the additional advantage of being able to deposit large quantities of drugs and proteins even to the inner parts of cells where certain drugs cannot reach.^74,75^ Normally, large-sized drugs can be expelled from the body easily by excretion, but the small size of NPs allows numerous drugs to be taken in by cells and persist for longer periods of time in the body. Moreover, they are able to penetrate deep into living tissue where they may need to perform certain functions.^67^ Such conjugate properties improve the effectiveness of medicines, especially for targeted therapy where drugs need to be delivered to certain sites that other drugs cannot reach under normal circumstances.^76^

GBM is categorised as the most dangerous cancer of the brain and usually develops from astrocytoma cells.^77^ The survival period is very short once diagnosed (approximately 90 days) and several drugs have been synthesized for GBM but have shown negative side effects during the post-treatment stage. It is cancers such as these that have motivated researchers to conjugate NPs and CTX to acquire satisfactory drug properties and overcome the challenge of multi-drug resistance.^78,79^ Of late, owing to advancements in drug delivery research, it has been confirmed that anticancer drugs can be improved several-fold when conjugated with NPs. Literature has shown and documented that NPs and CTX are very effective when conjugated for depositing drugs at specific sites, such as right inside the tumor and at a faster rate.^80^ Currently, quite a number of NP-CTX drugs such as methotrexate (MTX) are on trial and yielding promising results.^74,80^ More so, NPs have enabled oncologists to locate sentinel nodes for mapping and imaging during treatment. These NPs are able to detect and image tumor ascites, whereas other chemically synthesized particles are unable to do so. In addition, they are able to ‘tell’ the surgeons once an operation is complete through the observation of concentrated fluorescence in the lymph node site. The functions of certain hydrophilic NPs, such as chitosan, nano-gold, nano-silver, magnetic and super-magnetic NPs, as well as dendrimers, are being investigated thoroughly to determine their efficacy as vehicles for therapeutic and effective venoms, proteins, antigens and peptides.^12,81,82^

## Use of chlorotoxin in radiotherapy

Post-operative radiotherapy is a novel measure taken to improve the survival rates of patients after cancer treatment, but despite this, resultant death, every so often caused by cancer of the brain, is still frequently reported. One approach has been to increase dosimetry in the glioma cell to deposit the drug in the GBM. To improve this, the substance requires the ability to pass through the brain-blood barrier effortlessly and bind specifically to glioma cells so that the deposition of radiation to normal surrounding cells decreases.^83^ Radioactive iodine has been the main therapy used to cure thyroid cancer during problematic post-operative procedures to destroy almost insignificant amounts of thyroid cancer cells that escape treatment and later become problematic by remaining active as recurrent cancer. However, the technique has proven harmful to surrounding healthy cells.^83,84^ This has therefore elucidated CTX as a novel and better candidate in comparison to radiotherapy and other available therapies. Mamelak and Jacoby showed that CTX could easily penetrate small-sized channels because of its minute size.^85^ In addition, its excretion has been confirmed through a study by Dardevet et al, indicating that 90% of the peptide is excreted from the body within 24-36 hours, making CTX a suitable candidate with favourable properties for radiation.^24^

## Potential limitations of available diagnostic tools and delays in the early cure of cancer

Current attempts to combat cancer, which include primary prevention, early diagnosis and improved therapies, are not wholly successful mainly because the signs and symptoms associated with the disease delay and produce negative effects during late stages. The main questions to be addressed remain when and what interventions are needed to improve cancer therapies. One of the most assured ways is to improve early diagnosis and thus allow patients the opportunity to start treatment promptly and increase their chances of survival. Different biomarkers and diagnostic tools are currently in use, such as mass spectroscopy and surface-enhanced laser desorption/ionization-time-of-flight (SELDITOF) mass spectrometry. These devices are mainly used in clinical laboratories as diagnostic tools and are based on pre-treatment of biological fluids, binding of metals, ion exchange and other interactions. The bound proteins are exposed to mass spectrometric studies and the collected information is then used to detect and diagnose the disease. This approach, however, is limited by the use of fluids that include blood serum-containing proteins that upregulate during tumor formation of the tumor host microenvironment, which may not be enough to cause proteomic pattern changes used by SELDITOF technology. This, therefore, calls for the improvement of current techniques or the development of new ones that are ultrasensitive and able to detect changes in protein concentrations at very low doses (far lower than those achieved by currently available SELDITOF technologies). Even though many available biomarkers have been established (Table 1),^86^ they lack specificity, sensitivity, and predictability. It has now been reported that adolescents and young adults (aged 20-39 years) are most susceptible to cancer owing to lack of medical attention.^87-89^ This is, therefore, a wake-up call to consider promising natural compounds in the diagnosis and treatment of cancer to address such challenges.

**Table 1:** Currently available biomarkers designed for specific cancers. Table adapted from Kulasingam and Diamandis^86^

**Type of cancer biomarkers**	**Type of cancer**
Prostate-specific antigen (PSA)	Prostate cancer
Carcinoembryonic antigen (CEA)	Colon, lung, and breast cancer
Carbohydrate antigen (CA) 125	Ovarian cancer
CA 15-3	Breast cancer
CA 19-9	Gastrointestinal cancer
Alpha-fetoprotein (AFP)	Liver and testicular cancer
Human choriogonadotropin (hCG)	Testicular and gestational cancer

## Future prospects of chlorotoxin in cancer diagnostics and therapeutics

The early detection of cancer is an important step in cancer control and prevention. Despite previous breakthroughs in the improvement of cancer screening or detection, most techniques lack specificity and sensitivity, which may, in turn, result in late diagnosis where several pathological processes have already caused too much damage and have permitted the disease to relocate to other important organs and distal parts of the body. It has been observed in more than 60% of cancer patients that once the disease has metastasized, therapeutic drugs tend to lose their effectiveness.^90^ This then raises the need to detect cancer either during its pre-malignancy state or while in its very early stages to increase chances of total cure and survival, leading to the big question: how can cancer be detected during its early stages (i.e. the pathophysiological stage)? Although incredible progress has been made in understanding cancer at the molecular level, sizable gaps still need to be filled in understanding the pathogenesis of the disease as well as creating innovative strategies in drug development.

One of the biggest challenges facing cancer diagnostics is inability to screen for important proteins expressed at different cancer stages. Proteomics is fast becoming a useful tool in the field of biotechnology and aims to overcome current limited approaches in investigating diseases by using upregulated proteins in pathological processes or in examining damage to certain organs like the heart, lungs or kidneys to detect disease. By screening proteins expressed by these organs, new and improved drugs and biomarkers can be developed.^91^ Hence, proteomics can then be employed as a powerful tool by using CTX in the design of new and powerful diagnostic markers for cancer.^92,93^

Genetics can be identified as responsible for only a small percentage of the many cases and causes of cancer. This in itself conveys the need for a biomarker, a cellular indicator in both the physiological and pathological state that detects the onset of cancer at the molecular level of development. Moreover, the need to create diagnostic tools that can indicate the exact stages of cancer onset is vital. Current markers lack specificity, thus it is proposed that in future, CTX can and should be used to synthesize diagnostic markers to assist and improve the specificity and sensitivity of current diagnostic tools, since a lot of research has made evident that CTX is a highly specific peptide. A study by Veiseh and colleagues conjugated CTX with the molecular beacon Cy5.5 to produce CTX-Cy5.5, which produces fluorescence in a near-infrared spectrum. Using 50 mice, they showed that tissues infected with cancerous cells were clearly and successfully differentiated from normal histological cells when injected with CTX-Cy5.5. This showed the ability of CTX to improve specificity to cancerous cells, particularly to glioblastoma cells, in this case, for treatment.^69^ This study also demonstrated how CTX could be used as a modern device to synthesize biological prototypes that can be employed as diagnostic tools.

Over the last two decades, explosive breakthroughs related to different venoms have occurred in the world of research for their use in therapeutics and this has led to the design of numerous drugs from these substances. From studies conducted on CTX and the salient properties it has been proven to possess, as well as the potential biopharmaceutical applications it contains, different therapeutic drugs are now at different phases of development, which is indicative of the fact that peptides are now becoming more useful in the biopharmaceutical sector.^94^ The use of CTX comes as a breakthrough especially in the diagnostics of cancer. Literature has already hypothesized the interaction of CTX and MMP-2, a protein highly upregulated during tumor production. Therefore, understanding the mechanism by which these two proteins interact is crucial for the design of prototype biological devices or diagnostic kits similar to HIV-screening kits, where CTX can be mobilised onto a solid surface and exposed to blood samples of individuals to ascertain the presence or up-regulation of certain proteins (e.g. MMP-2) during the development of tumors. Therefore, the identification of MMP-2 as a drug target would be a vital step in diagnostics.^95-97^ In this way, the presence of tumors, whether malignant or not, can be screened and identified at a very early stage, probably through the observance of colour change, and thus, proper precautions could be taken. However, CTX and MMP-2 interaction has only been hypothesized, hence it would be of the essence not only to establish the interaction between the two proteins, but also to determine the thermodynamic properties of the interaction. Such a diagnostic tool would not only be easy to use in non-clinical settings, but would also be portable and allow for immediate screening to take place at any time and anywhere.^98^

## Effect of CTX on renal and liver function

There has been growing interest in recent years for the discovery and development of naturally-produced therapeutics from plants or animals that not only function as anti-cancer drug delivery carriers or vectors but also as compounds that can easily be degraded by the human system and excreted at a fast rate.^25,99^ CTX has attracted attention as a possible colloidal drug delivery system that seeks to overcome the unfavourable physical and chemical features of synthetic drugs. In an endeavour to use CTX as a ‘theranostic’ agent, CTX was attached to radioactive iodine (^125^I and ^131^I) as an anti-tumor radiotherapy agent with the ability to either locate the position of the tumor through imaging or determine tumor volume or physiology.^100-102^ Results showed ^125^I-CTX CTX could facilitate cancer-cell identification in biopsies from human glioma patients, while other outcomes of the experiment led to the development of ^131^I-CTX into a therapeutic delivery payload. Ultimately, it was shown that although accumulation was observed in the stomach and thyroid of ^125^I-CTX administered mice, it could be cleared from the body through the urinary system, while ^131^I-CTX, on the other hand, was well tolerated and cleared within 24-48 hours. Moreover, no organ failure was reported, thus indicating that CTX does not interfere with the normal liver function. However, Phase II of the study has yet to be conducted. In other studies, the bioconjugation of CTX and Cy5.5 by Veiseh and colleagues^69^ as an imaging agent for glioma, prostate and intestinal cancer, medullablastoma and sarcoma mouse models was done. Evidence of renal excretion was observed by the collection of the compound in the renal system as is typically expected of compounds to be excreted through urine. Additionally, no evidence of cytotoxicity was observed after administration of the bioconjugate to the mice even after two weeks of exposure.^24^ These studies show the ability of CTX not only to be broken down but also cleared from the system after performing its function.

## CTX interaction with other drugs

MTX is a commercially used chemotherapeutic drug for the treatment of neck and head cancer, leukaemia and osteosarcoma. MTX functions through cytotoxicity as a folate pathway antagonist, stimulating competitive inhibition of the enzyme dihydrofolate reductase, which in turn inhibits deoxythymidine monophosphate and purine precursors that facilitate DNA synthesis.^103^ However, reported drawbacks of the drug include its short blood half-life and the toxic side effects associated with its administration. In an experiment by Sun and colleagues,^103^ CTX was bounded to MTX and a polyethylene glycol layer as a coat and ligand-linking molecule. This supramolecular compound was thereafter bioconjugated to iron oxide nanoparticles (NP-MTX-CTX) as a tumor targeting nanovector for both therapeutics and diagnostics to circumvent the shortcomings of MTX. Cell viability tests showed an increase in cancer cell cytotoxicity by the NP-MTX-CTX system in comparison to treatment with NPs and MTX only, hence showing that CTX improved MTX effectiveness.^103^ Additionally, accumulation of the conjugate was observed more in cancerous cells than in normal healthy cells through magnetic resonance imaging.^103^ The experiment also highlighted its success in the development of a system capable of delivering chemotherapeutic agents to the tumor cells with high specificity and its ability to be retained in the tumor at least for a minimum of two weeks.^103^ Wang and colleagues^104^ in a similar study, synthesized and characterized chlorotoxin-conjugated graphene oxide (CTX-GO) nanosheets whose two–dimensionally large aromatic surface and abundant functional groups permitted its use in research as an anti-tumor drug delivery system.^104^ In the study, doxorubicin, a chemotherapy agent was successfully loaded non-covalently with high efficiency onto the chlorotoxin-bioconjugated graphene oxide sheets (CTX-GO/DOX) and used to treat C6 glioma cells. Results showed delivery of doxorubicin to the cells was pH-dependent, while *in vitro* cytotoxicity assays showed that in comparison to free doxorubicin and GO/DOX, the conjugation of CTX was the possible factor that allowed for the higher rate of glioma cell death observed during the experiment.

Onconase, though not a drug, is a 104 amino acid and four disulphide bonded RNase that has shown anti-tumor potential by degrading RNA and causing cancer cell death. Studies by Wang and Guo^105^ took advantage of the glioma targeting and cytotoxicity properties of CTX and Onc respectively by using the disulphide linkages to covalently conjugate recombinant CTX to recombinant Onc (CTX-Onc) to treat cultured U251 and SGH-44 glioma cells, as well as tumorous nude mouse models. Results revealed higher cytotoxicity and better anti-tumor effect using the CTX-Onc conjugate than the physical mixture of the CTX and Onc (CTX + Onc) control.^105^ All these studies show the potential CTX possesses in enhancing currently available drugs for improved chemotherapy agents.

## Conclusion

**Figure 1:**
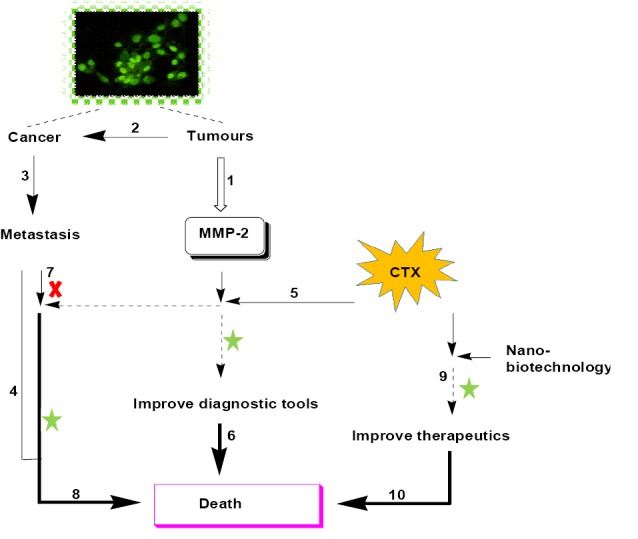
**Proposed model of CTX functioning and its use in cancer biology.** In the model, the following processes are vividly elucidated: (1) the upregulation of MMP-2 proteins from the extracellular matrix of tumors; (2) pathophysiological process, where tumors become invasive cancer cells; (3) cancer cells undergoing metastasis to distal parts of the body; (4) uncontrolled metastasis leading to death; (5) CTX’s ability to bind to upregulated MMP-2; (6) improvement of cancer diagnostic tools to delay death; (7) binding of CTX to MMP-2 delays the metastasis process; (8) reduced metastasis benefits patient life span; (9) CTX conjugated with NPs in nano-biotechnology, to (10) improve cancer therapeutics with the main aim of improving patient life and preventing early death.

The aim of this review was to display the many and different properties of CTX that have potential use in biomedical applications, with special attention being paid to cell imaging, radiotherapy and medications conjugated to NPs for cancer therapeutics and diagnostics. It appears that the use of venoms such as CTX to design more effective drugs and diagnostic tools could easily turn out to be a ‘golden’ approach for the screening of tumors at very early stages of development.^106^ Figure 1 shows a proposed model for future CTX applications for the synthesis of biological devices at early pathophysiological stages, even before the onset of the clinical features of cancer. This model additionally illustrates how the early application or use of CTX can be highly beneficial, not only for early cancer detection, but also for post-cancer treatment, thereby prolonging survival. Unfortunately, many clinical features, signs or symptoms of cancer may, in many cases, be delayed and only become evident when it is already too late. Achieving early detection, therefore, calls for intense biochemical studies to be conducted towards the development of an early point-of-care diagnostic kit or tool. This will be extremely useful, particularly in neglected and rural areas where specialized clinical equipment and medication are not only expensive but also scarce. In addition, most patients are only hospitalized once visible symptoms have manifested themselves and the disease has already caused irreversible damage. Although this review leaned towards the many positive properties of CTX, there is still a need for additional and extensive research into its biological mechanism and how it may be used in the suggested tools.

## Ethical Issue

Not Applicable.

## Conflict of Interest

Authors declare no conflict of interest in this study.
